# Palladium-Mediated Synthesis of [Carbonyl-^11^C]acyl Amidines from Aryl Iodides and Aryl Bromides and Their One-Pot Cyclization to ^11^C-Labeled Oxadiazoles

**DOI:** 10.1021/acs.joc.2c02102

**Published:** 2022-12-13

**Authors:** Jonas Rydfjord, Silav Al-Bazaz, Sara Roslin

**Affiliations:** Department of Medicinal Chemistry, Uppsala University, BMC Box 574, SE-751 23 Uppsala, Sweden

## Abstract

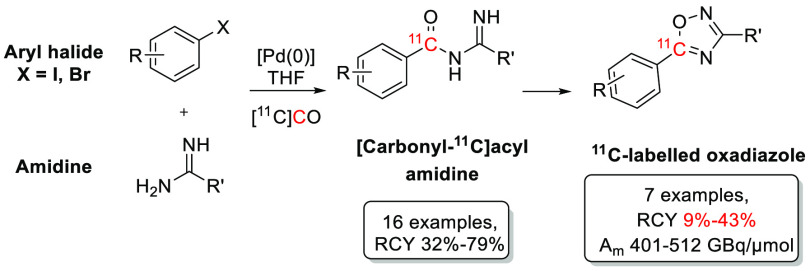

Positron emission tomography (PET) is a highly valuable imaging technique with many clinical applications. The possibility to study physiological and biochemical processes in vivo also makes PET an important tool in drug discovery. Of importance is the possibility of labelling the compound of interest with a positron-emitting radionuclide, such as carbon-11. Carbonylation reactions with [^11^C]carbon monoxide ([^11^C]CO) has been used to label a number of molecules containing a carbonyl derivative, such as amides and esters, with carbon-11. Presented herein is the palladium-mediated carbonylative synthesis of [carbonyl-^11^C]acyl amidines and their subsequent cyclization to ^11^C-labeled 1,2,4-oxadiazoles. Starting from amidines, [^11^C]CO, and either aryl iodides or aryl bromides, [carbonyl-^11^C]acyl amidines were synthesized and isolated in good to very good radiochemical yields (RCY). The ^11^C-labeled 1,2,4-oxadiazoles were synthesized without the isolation of the intermediate [carbonyl-^11^C]acyl amidines and isolated in useful RCYs, including the NF-E2-related factor 2 activator DDO-7263. 3-Phenyl-5-(4-tolyl)-1,2,4-(5-^11^C)oxadiazole was synthesized and isolated with a clinically relevant molar activity. The broadened substrate scope, together with the good RCY and high *A*_m_, demonstrates the utility of this method for the incorporation of carbon-11 into acyl amidines and 1,2,4-oxadiazoles, structural motifs of pharmacological interest.

## Introduction

Positron emission tomography (PET) is a noninvasive imaging technique that utilizes short-lived positron emitting radionuclides such as carbon-11 (*t*_1/2_ = 20.4 min) and fluorine-18 (*t*_1/2_ = 109.8 min) to study physiological and biochemical processes in vivo. PET has become an important tool for clinicians, with applications in cardiology, neurology, and oncology.^1−4^ The possibility of gaining insight into molecular mechanisms makes the PET technique an important tool in both preclinical and clinical research.^5,6^ Central to utilizing the PET technique is thus the ability to incorporate a positron-emitting radionuclide in the compound of interest. The continued development of new radiochemical labeling methods helps prevent the labeling step from being the limiting factor with clinically and preclinically interesting compounds.^7−9^

**Figure 1:**
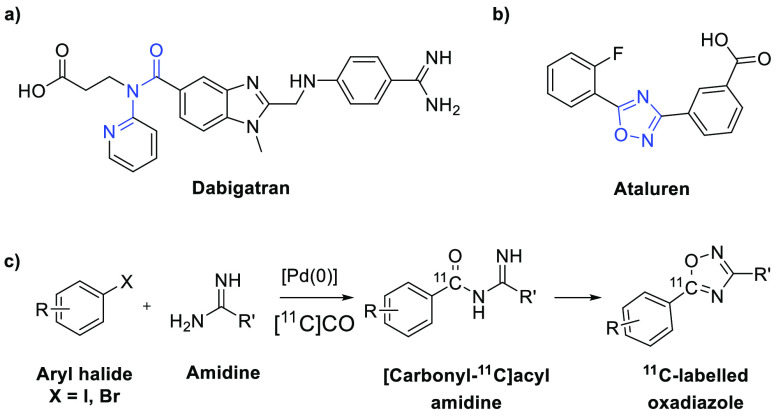
(a) Dabigatran, a thrombin inhibitor. The acyl amidine motif is highlighted. (b) Ataluren, a drug that promotes nonsense suppression. The 1,2,4-oxadiazole motif is highlighted. (c) The reaction presented herein.

Acyl amidines and 1,2,4-oxadizoles are both interesting from a drug discovery perspective.^10^ The acyl amidine motif is found in pharmacologically active compounds such as renin inhibitors,^11^ angiotensin II receptor ligands,^12^ thrombin,^13^ β-secretase,^11^ and cathepsin D inhibitors^11^ (see Figure 1a). Aside from being interesting structures, acyl amidines are used as precursors to different heterocycles such as 1,2,4-oxadiazoles,^14,15^ 1,3,5-triazines,^16^ 1,2,4-triazoles,^17,18^ and 1,2-dihydro-3*H*-pyrrol-3-ones.^19^ The 1,2,4-oxadiazole motif is found in compounds with pharmacological activity,^20^ such as ataluren,^21^ a drug that promotes nonsense suppression, DDO-7263,^22^ an NF-E2-related factor 2 (Nrf2) activator, and antitumoral compounds^23^ (see Figure 1b). The oxadiazole ring also serves as a bioisostere for esters and amides.^24^ These varied biological activities make acyl amidines and the 1,2,4-oxadiazoles structures of interest for carbon-11 labeling, thus presenting an option to study promising compounds with these structural motifs with PET.

Palladium and other transition metals have been widely used as catalysts together with carbon monoxide or carbon monoxide surrogates in the synthesis of various heterocycles.^25−27^ Apart from the richness in the types of readily available metal catalysts, there is also a large variation in the types of starting materials that can be utilized for carbonylative heterocycle synthesis.^28−32^ In a paper published in this issue, the palladium-catalyzed synthesis of acyl amidines from aryl halides and amidines and their subsequent cyclization to form 1,2,4-triazoles and 1,2,4-oxadiazoles was explored.^33^ In that paper, the synthetic protocol was adapted to synthesize carbon-11-labeled acyl amidines and 1,2,4-oxadiazoles on a small scale. To the best of our knowledge, this constituted the first reported carbon-11 labeling of acyl amidine and the 1,2,4-oxadiazole ring scaffold. Through a palladium-mediated reaction in 1,4-dioxane, aryl iodides and amidines afforded [carbonyl-^11^C]acyl amidines, which could be cyclized to ^11^C-labeled 1,2,4-oxadiazoles without the isolation of the intermediate [carbonyl-^11^C]acyl amidine. The successful adaption of a two-step, one-pot approach is exciting because it provides another route to carbon-11-labeled heterocycles through the use of [^11^C]carbon monoxide instead of other carbon-11 precursors such as [^11^C]nitromethane,^34^ [^11^C]carbon dioxide,^35^ and [^11^C]phosgene.^36^

In the present study, we sought to improve the previously described palladium-mediated synthesis of [carbonyl-^11^C]acyl amidines and their subsequent one-pot cyclization to form ^11^C-labeled oxadiazoles as well as to extend the reaction to include the use of aryl bromides as starting materials for the formation of [carbonyl-^11^C]acyl amidines (see Figure 1c).

## Results and Discussion

### [Carbonyl-^11^C]acyl Amidines

To optimize the synthesis of [carbonyl-^11^C]acyl amidines from aryl iodides, 4-iodotoluene (**1a**) and benzamidine (**2a**) were chosen as model starting materials for the synthesis of *N*-(imino(phenyl)methyl)-4-methylbenzamide (**3a**). 1,4-Dioxane and palladium tetrakis(triphenyl-phosphine) were used in the synthesis of [carbonyl-^11^C]acyl amidines in Rydfjord et al.,^33^ but here the solvent was changed to tetrahydrofuran for the optimization experiments. The catalyst and the reaction temperature were evaluated as shown in Table 1. The amount of [^11^C]CO trapped in the reaction vial is a measurement of how well the gaseous [^11^C]CO was incorporated in a nonvolatile reaction product and was in general very high (≥98%) for the different conditions tested. On the other hand, the product selectivity varied, as determined by HPLC analysis of the crude reaction mixture. Most of **3a** was formed (95%) with the conditions in entry 1, where Pd(PPh_3_)_4_ was used and the reaction mixture was heated to 120 °C. Using either palladium acetate and triphenylphosphine (entry 2) or heating the reaction at 150 °C (entry 3) gave lower yields (76% and 73%, respectively) due to byproduct formation. Changing the solvent from 1,4-dioxane to THF but keeping the temperature and the catalyst as in the paper copublished in this issue^33^ dramatically improved both the trapping of [^11^C]CO and the product selectivity, increasing the nonisolated yield from 54% to 95%.

**Table 1:**
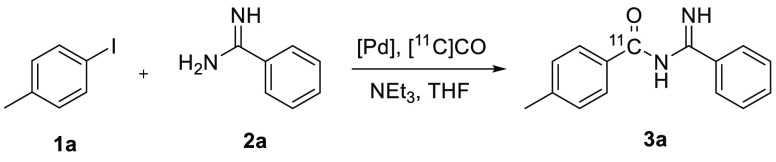
Optimization of the Synthesis of [Carbonyl-^11^C]acyl Amidines from 4-Iodotoluene^a^

entry	catalyst	temp (°C)	[^11^C]CO trapped in solution (%)^b^	product selectivity^c^ (%)	nonisolated RCY^d^ (%)
**1**	Pd(PPh_3_)_4_	120	98 ± 2	97 ± 3	95 ± 4
**2**	Pd(OAc)_2_/PPh_3_	120	98 ± 1	73 ± 4	76 ± 7
**3**	Pd(PPh_3_)_4_	150	98 ± 1	74 ± 4	73 ± 4

a Conditions: **1a** (2.0 mg, 9 μmol), **2a** (2.2 mg, 2 equiv), [Pd] (Pd(OAc)_2_ (0.5 mg, 0.25 equiv) or Pd(PPh_3_)_4_ (1.0 mg, 0.1 equiv)), PPh_3_ (1.2 mg, 0.5 equiv), NEt_3_ (5 μL, 4 equiv), and THF (400 μL). The reaction time was 10 min. Experiments were performed in triplicate.

b Decay-corrected.

c From HPLC-analysis of the crude reaction mixture.

d Estimated by calculating the sum of the trapped [^11^C]CO and the product selectivity.

To optimize the synthesis of [carbonyl-^11^C]acyl amidines from aryl bromides, 4-bromotoluene (**1i**) was chosen together with **2a** for the synthesis of **3a**. See Table 2 for the results. The conditions that were successful with aryl iodide **1a** (see Table 1) did not give any product when aryl bromide **1i** was used as the starting material (entry 1). The addition of xantphos as an additional ligand dramatically improved the trapping of [^11^C]CO in nonvolatile reaction products as well as the formation of **3a** (entry 2). Xantphos has previously proved successful in other types of carbonylation reactions with aryl bromides as starting materials.^33,37,38^ Next, the palladium catalyst was changed (entries 3–5). Here, dipalladium(π-cinnamyl)dichloride (Pd_2_(π-cinnamyl)Cl_2_) in combination with xantphos gave the best results for both trapping and product selectivity, thus giving an excellent nonisolated radiochemical yield of 92%. The successful combination of Pd_2_(π-cinnamyl)Cl_2_ and xantphos has been demonstrated for ^11^C-carbonylation reactions^39^ and was kept for the rest of the experiments. Instead, the choice of temperature and solvent were investigated. Raising the temperature to 150 °C proved to be disadvantageous, as the product selectivity decreased from 93% to 75% (entry 6). Likewise, using 1,4-dioxane or *N,N*-dimethylformamide (DMF) as solvent at either 120 or 150 °C increased the formation of side products and thus lowered the product selectivity, giving nonisolated RCYs of 38%–49% (entries 7–10) even though the trapping of [^11^C]CO was high (>89%) for all conditions tested.

**Table 2:**
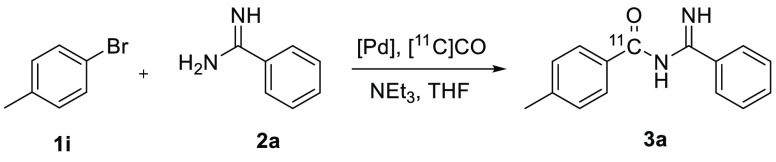
Optimization of the Synthesis of [Carbonyl-^11^C]acyl Amidines from 4-Bromotoluene^a^

entry	catalyst	temp (°C)	solvent	[^11^C]CO trapped in solution (%)^b^	product selectivity^c^ (%)	nonisolated RCY^d^ (%)
1	Pd(PPh_3_)_4_	120	THF	14 ± 6	N.D.	N.D.
2	Pd(PPh_3_)_4_/xantphos	120	THF	95 ± 8	79 ± 6	76 ± 11
3	Pd(OAc)_2_/xantphos	120	THF	74 ± 16	58 ± 8	43 ± 10
4	Pd_2_(dba)_3_/xantphos	120	THF	96 ± 1	75 ± 8	72 ± 9
5	Pd_2_(π-cinnamyl)Cl_2_/xantphos	120	THF	99 ± 1	93 ± 5	92 ± 4
6	Pd_2_(π-cinnamyl)Cl_2_/xantphos	150	THF	>99	75 ± 2	75 ± 2
7^e^	Pd_2_(π-cinnamyl)Cl_2_/xantphos	120	1,4-dioxane	89 ± 15	48 ± 21	45 ± 23
8	Pd_2_(π-cinnamyl)Cl_2_/xantphos	150	1,4-dioxane	>99	49 ± 2	49 ± 2
9	Pd_2_(π-cinnamyl)Cl_2_/xantphos	120	DMF	97 ± 2	40 ± 1	39 ± 2
10	Pd_2_(π-cinnamyl)Cl_2_/xantphos	150	DMF	99 ± 1	39 ± 8	38 ± 9

a N.D. = not determined. Conditions: **1i** (1.5 mg, 9 μmol), **2a** (2.2 mg, 2 equiv), Pd source (0.1 equiv), xantphos (0.2 equiv with monopalladium catalysts, 0.4 equiv with bipalladium catalysts), NEt_3_ (5 μL, 4 equiv), and solvent (400 μL). The reaction time was 5 min. Experiments were performed in triplicate.

b Decay-corrected.

c From HPLC analysis of the crude reaction mixture.

d Estimated by calculating the sum of the trapped [^11^C]CO and the product selectivity.

e Data from four experiments.

**Figure 2:**
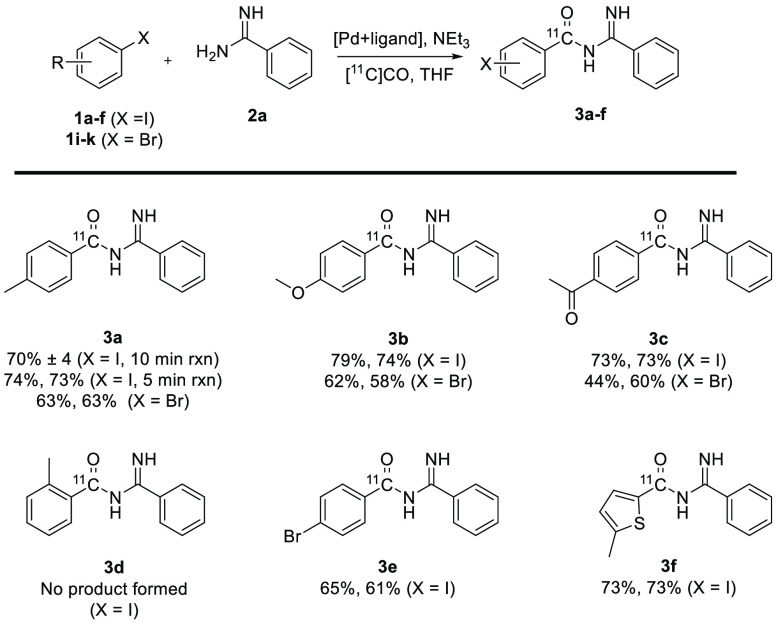
Scope of aryl halides in the synthesis of [carbonyl-^11^C]acyl amidines. Conditions are the same as those in either entry 1 of Table 1 for aryl iodides with a reaction time of 5 min unless stated otherwise or entry 5 of Table 2 for aryl bromides. Radiochemical yields are decay-corrected.

The optimized conditions for the synthesis of [carbonyl-^11^C]acyl amidine **3a** from either 4-iodotoluene (**1a**) or 4-bromotoluene (**1i**) were then used to explore the versatility of the reaction. Shown in Figure 2 is the synthesis of [carbonyl-^11^C]acyl amidines **3a**–**f** from various aryl iodides and aryl bromides.

The reaction worked very well for both aryl iodides and aryl bromides, with radiochemical yields for the isolated [carbonyl-^11^C]acyl amidines in general over 60%, peaking at a 79% radiochemical yield for **3b**. **3a** was synthesized from aryl iodide **1a** with both 5 and 10 min reaction times. The radiochemical yield did not improve with the longer reaction time (yields are decay-corrected back to the same point in time); rather, there was a small tendency for decreased product formation. The aryl iodides gave somewhat higher radiochemical yields compared with the aryl bromides (**3a**–**c**), but electron-poor and electron-rich aryl halides were found to perform about equally well, with a slight tendency for the electron-rich substrates to give higher yields. With the lack of reactivity for the aryl bromide under the reaction conditions optimized for aryl iodides (see Table 2, entry 1), 1-bromo-4-iodobenzene could be utilized as a starting material in the synthesis of **3e**. The limitation of the reaction came with the attempted synthesis of **3d**. The *ortho*-substituted product could not be detected. The palladium and ligand sources were changed to Pd(OAc)_2_ and DPEPhos ((oxidi-2,1-phenylene)bis(diphenylphosphine)), respectively, which had worked well in the synthesis with carbon monoxide,^33^ but **3d** could not be detected.

**Figure 3:**
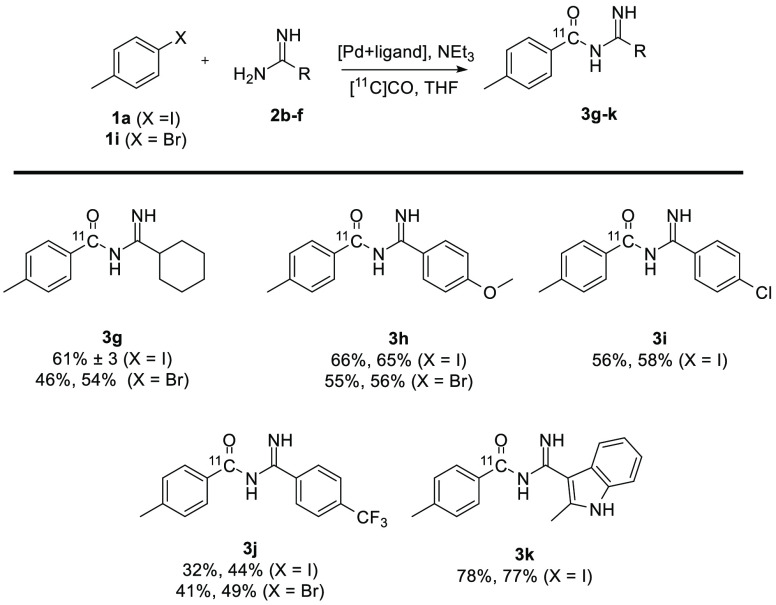
Scope of amidines in the synthesis of [carbonyl-^11^C]acyl amidines. Conditions are the same as those in either entry 1 of Table 1 with **1a** as the aryl halide or entry 5 of Table 2 with **1i** as the aryl halide. Radiochemical yields are decay-corrected.

Next, the scope of the amidines in the synthesis of [carbonyl-^11^C]acyl amidines was explored, as shown in Figure 3. The radiochemical yields were generally high but varied a bit, with **3k** being isolated with a 78% yield and **3j** isolated with a 32% yield. The same trend of aryl iodides performing slightly better than their aryl bromide counterparts could be seen (**3g** and **3h**), although in the synthesis of **3j** aryl bromide **1i** gave higher yields (41% and 49% compared to 32% and 44%). The electron-rich amidines tended to perform better than the electron-poor amidines (**3h** and **3k** compared to **3i** and **3j**) in reactions with **1a**.

### ^11^C-Labeled Oxadiazoles

With the optimized conditions for the synthesis of [carbonyl-^11^C]acyl amidines in hand, their cyclization to ^11^C-labeled oxadiazoles was explored next. The optimization of the cyclization reaction starting with 4-iodotoluene (**1a**) and 4-bromotoluene (**1i**) can be seen in Tables 3 and 4, respectively. Hydroxylamine hydrochloride dissolved in 50% acetic acid (aq) was used to facilitate the cyclization reaction. The cyclization temperature was set to 150 °C, the same as that in Rydfjord et al.^33^

**Table 3:**
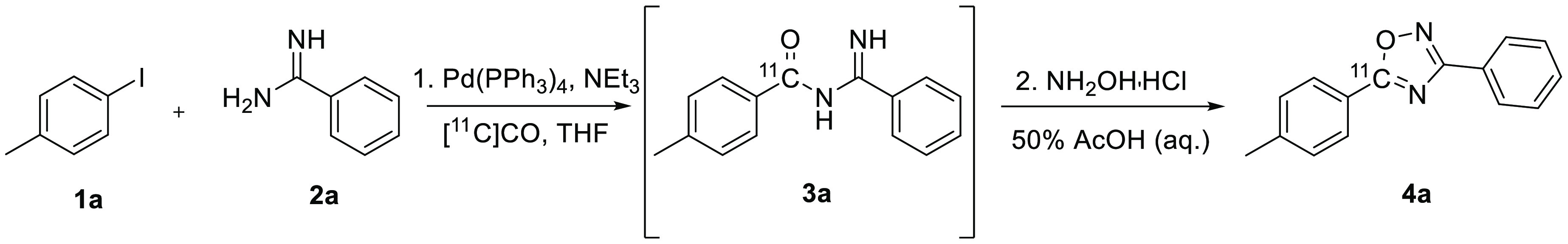
Optimization of the One-Pot, Two-Step Synthesis of ^11^C-Labeled Oxadiazoles from Aryl Iodides^a^

entry	temp. increase (min)	cyclization time (min)	[^11^C]CO trapped in solution (%)^b^	product selectivity^c^ (%)	nonisolated RCY^d^ (%)
1	4.5	5	95 ± 3	41 ± 11	39 ± 10
2	4	5	93 ± 5	53 ± 5	49 ± 4
3	4	10	49 ± 32	36 ± 16	21 ± 20
4^e^	4	5	94 ± 6	53 ± 1	50 ± 4

a Conditions: (1) **1a** (2.0 mg, 9 μmol), **2a** (2.2 mg, 2 equiv), Pd(PPh_3_)_4_ (1.0 mg, 0.1 equiv), NEt_3_ (5 μL, 4 equiv), THF (400 μL), reaction temperature of 120 °C, and 5 min reaction time. (2) NH_2_OH·HCl (4.4 mg, 7 equiv) dissolved in 50% acetic acid (aq) and reaction temperature of 150 °C. Experiments were performed in triplicate.

b Decay-corrected.

c From HPLC analysis of the crude reaction mixture.

d Estimated by calculating the sum of the trapped [^11^C]CO and the product selectivity.

e The reaction was performed using 14 equiv of NH_2_OH·HCl.

**Table 4:**
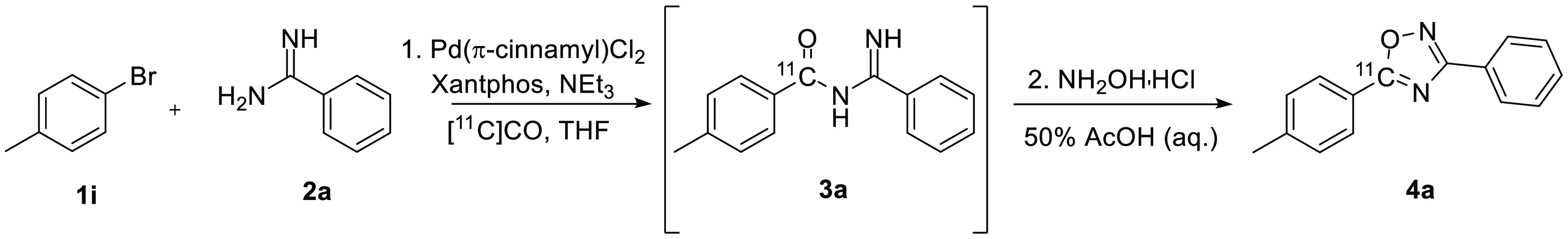
Optimization of the One-Pot, Two Step Synthesis of ^11^C-Labeled Oxadiazoles from Aryl Bromides

entry	solvent	reaction temperature step 1 (°C)	[^11^C]CO trapped in solution^b^ (%)	product selectivity^c^ (%)	nonisolated RCY^d^ (%)
**1**	THF	120	99 ± 1	32 ± 5	32 ± 5
**2**	DMF	120	98 ± 1	5 ± 2	5 ± 2
**3**	THF	150	99 ± 0	19 ± 6	19 ± 6

a Conditions: (1) **1i** (1.5 mg, 9 μmol), **2a** (2.2 mg, 2 equiv), Pd(π-cinnamyl)Cl_2_ (0.5 mg, 0.1 equiv), xantphos (1.0 mg, 0.2 equiv of NEt_3_ (5 μL, 4 equiv), solvent (400 μL), reaction time of 5 min. (2) NH_2_OH·HCl (4.4 mg, 7 equiv) dissolved in 50% acetic acid (aq) and reaction temperature of 150 °C. In entries 1 and 2, the temperature was increased to 150 °C after 4 min.

b Decay-corrected.

c From HPLC analysis of the crude reaction mixture.

d Estimated by calculating the sum of trapped [^11^C]CO and the product selectivity.

In Table 3, entry 1, the temperature was raised to 150 °C 4.5 min into the synthesis of **3a** and the cyclization time was set to 5 min. **3a** was not isolated before the addition of hydroxylamine hydrochloride. The trapping of [^11^C]CO in the nonvolatile reaction products was nearly quantitative, and the selectivity for formation of **4a** was 41%, giving a nonisolated radiochemical yield of 39%. Next, the temperature was increased 4 min into the synthesis of **3a**, which increased the product selectivity (53%) and therefore raised the nonisolated radiochemical yield to 49% (entry 2). Increasing the cyclization time to 10 min was not advantageous for the reaction, as both the trapping and the product selectivity decreased, the former possibly due to the septum leaking and the latter due to the formation of byproducts (entry 3). Lastly, increasing the amount of hydroxylamine hydrochloride in entry 4 from 7 to 14 equiv gave results similar to those in entry 2. Thus, the conditions in entry 2 were used to explore the scope of the cyclization reaction.

The optimization of the cyclization reaction starting with 4-bromotoluene started from the conditions in entry 2 of Table 3. Performing the cyclization reaction as in entry 2 of Table 3 with **1i** as starting material gave a 32% nonisolated yield (entry 1, Table 4). Changing the solvent from THF to DMF proved to be deleterious for oxadiazole formation (entry 2), although DMF has been successfully used in a similar reaction.^14^ Performing both reaction steps at 150 °C was also not advantageous (entry 3). The conditions in entries 2 and 3 gave more byproducts compared to the conditions in entry 1, which then was used to explore the scope of the cyclization reaction. Of note is that around 12–20% of acyl amidine **3a** was left in the oxadiazole synthesis that started from 4-iodotoluene **1a**, but no traces of **3a** could be found in the HPLC chromatograms of the crude reaction mixtures that started from 4-bromotoluene **1i**.

**Figure 4:**
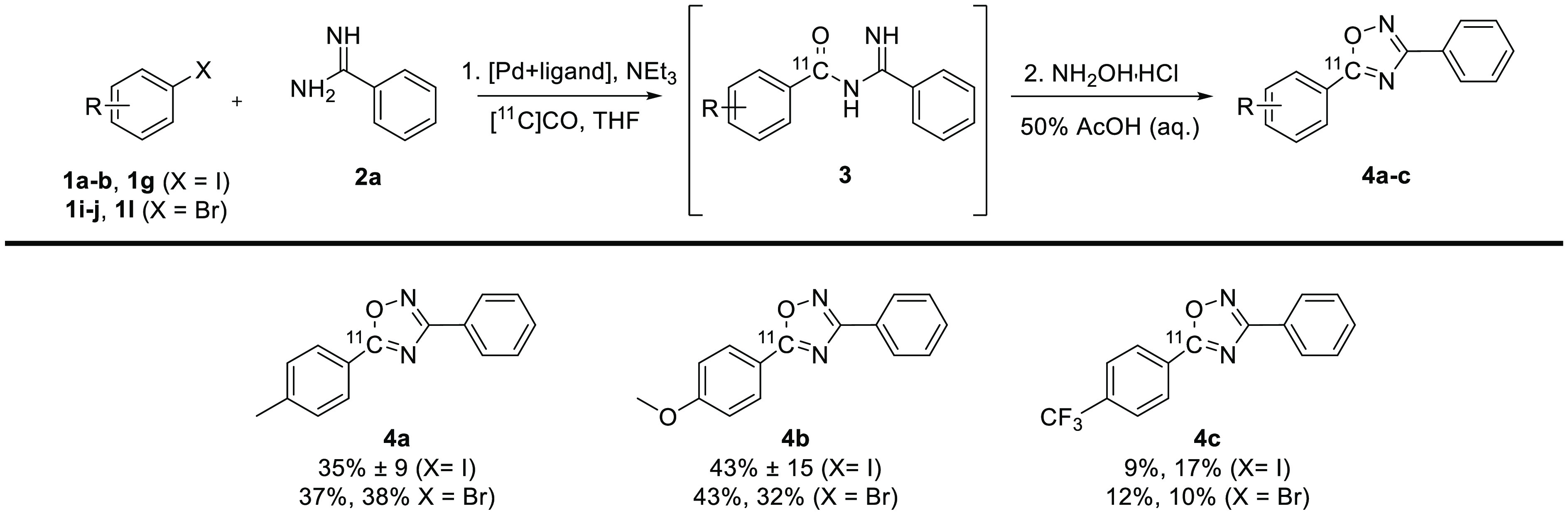
Scope of aryl halides in the synthesis of ^11^C-labeled oxadiazoles. Conditions are the same as those in entry 2 of Table 3 for aryl iodides and the same as those in entry 1 of Table 4 for aryl bromides. Radiochemical yields are decay-corrected.

A small set of ^11^C-labeled oxadiazoles was synthesized starting from aryl iodides **1a**, **1b**, and **1g** or aryl bromides **1i**, **1j**, and **1l** and amidine **2a** (Figure 4). The isolated radiochemical yields were in the range of 9–43%, with markedly higher yields for reactions with the toluene derivatives **1a** and **1i** or electron-rich anisole derivatives **1b** and **1j** (32%–43%) compared to the electron-poor derivatives **1g** and **1l**. In contrast to the synthesis of the [carbonyl-^11^C]acyl amidines, where there was a clear tendency for higher yields in reactions started from aryl iodides, no such difference could be seen in the synthesis of ^11^C-labeled oxadiazoles. It thus seems that the amount of [carbonyl-^11^C]acyl amidine does not determine the rate at which the ^11^C-labeled oxadiazole cyclizes.

**Figure 5:**
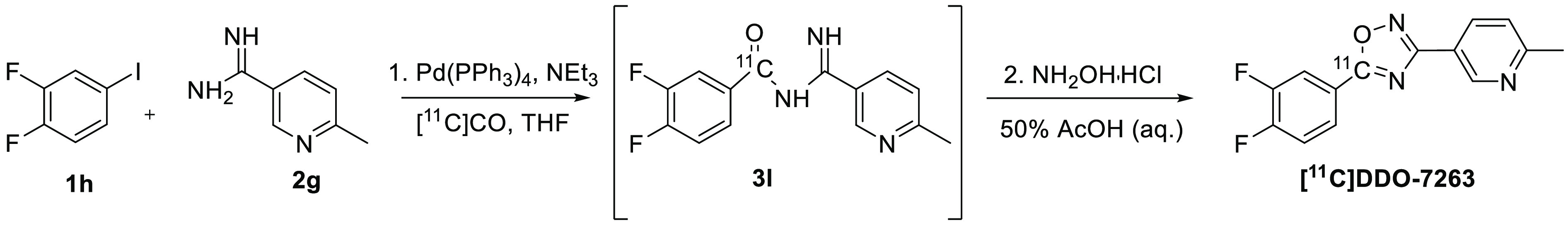
Synthesis of Nrf2-activator DDO-7263 labeled with carbon-11 in position 5 of the oxadiazole ring. Conditions are the same as those in entry 2 of Table 3.

1,2,4-Oxadiazole Nrf2-activator DDO-7263, which to the best of our knowledge was not previously labeled with carbon-11, was then synthesized (Figure 5). Starting with aryl iodide **1h** and amidine **2g**, [^11^C]DDO-7263 could be synthesized and isolated in a 21% RCY. DDO-7263 has demonstrated a therapeutic effect in 1-methyl-4-phenyl-1,2,3,6-tetrahydropyridine (MPTP)-induced Parkinson’s disease in mice by targeting Nrf2, a regulator of cellular redox homeostasis.^22,40^

### Molar Activity Determination

The molar activity describes the amount of radioactivity per the amount of unlabeled compound (in this case, all carbons are carbon-12) and is typically presented as gigabecquerel per micromole. A high molar activity is desirable, as this decreases the amount of unlabeled compound administered during a clinical or preclinical PET scan. The molar activity is hence an important part of the PET microdosing concept, a regulatory concept that facilitates first-in-man studies.^41−43^ The key part is the administration of very low doses of a compound labeled with a radioactive nuclide for imaging with highly sensitive equipment, such as a PET camera. Because of the low natural abundance of carbon monoxide in air, reactions with [^11^C]CO typically give labeled products with a high molar activity.

The molar activity was determined for **4a** after two irradiations of around 20 μAh. Starting from 24.7 GBq of [^11^C]CO, **4a** was isolated with 2.6 GBq of [^11^C]CO 35 min after the start of the synthesis, giving a molar activity of 512 GBq/μmol. In the second experiment, the synthesis started with 16.8 GBq of [^11^C]CO, and **4a** was isolated with 1.3 GBq of [^11^C]CO after 33 min, giving a molar activity of 401 GBq/μmol (please see the Supporting Information for all calculation details). These high molar activities are in line with previous syntheses with [^11^C]CO.^44,45^

## Conclusion

Presented herein is an improved protocol for the synthesis of [carbonyl-^11^C]acyl amidines and their cyclization to form ^11^C-labeled oxadiazoles. The protocol utilizes different substrates, including heteroaromatic substrates, resulting in consistently high radiochemical yields of the isolated [carbonyl-^11^C]acyl amidines. By changing the palladium source, the scope of the reaction could be widened to use aryl bromides as reactants. The simple one-pot, two-step synthesis and isolation of ^11^C-labeled oxadiazoles in moderate to good yields, as in the unprecedented ^11^C-labeling of Nrf2-activator DDO-7263, nicely demonstrates the strength of carbonylation reactions to produce rather complex structures from relatively simple starting materials.

## Experimental Section

### General Information

Reagents and solvents were commercially available and used without further purification. Carbon-11 was prepared by a ^14^N(p,α)^11^C nuclear reaction using 17 MeV protons produced by a Scanditronix MC-17 cyclotron at PET Centre, Uppsala University Hospital, and obtained as [^11^C]carbon dioxide. The target gas used was nitrogen (AGA Nitrogen 6.0) containing 0.05% oxygen (AGA Oxygen 4.8). [^11^C]Carbon monoxide was prepared from [^11^C]carbon dioxide with a carbon monoxide system build in-house (hereafter referred to as the xenon system) and used as a reagent in the acyl amidine and heterocycle syntheses.^46^ Semipreparative purification of ^11^C-labeled compounds was performed on an Agilent Infinity system with a 1260 Infinity II quaternary pump, a 1260 Infinity II variable wavelength detector, and a Bioscan flowcount radiodetector coupled with an Universal interface box. The HPLC was equipped with ACE 5 C18-HL (5 μm, 250 × 10.0 mm) column, and 0.1% trifluoroacetic acid (aq) and acetonitrile were used as eluents. The identities, concentrations, and radiochemical purities of the purified ^11^C-labeled compounds were determined with an Agilent Infinity system (1260 Infinity II pump, 1290 Infinity II vial sampler, G7130A column oven, and 1290 Infinity II variable wavelength detector or 1290 Infinity II diode array detector in series with a Bioscan β^+^-flowcount radiodetector coupled with an Universal interface box) equipped with either a Merck Chromolith Performance RP-18e column (4.6 × 100 mm) or a Phenomenex Kinetex C18 2.6 μm (4.6 × 100 mm) column, and ammonium formate buffer (pH 3.5) and acetonitrile were used as eluents. Isotopically unmodified compounds were used as references. The procedures used for the synthesis of the reference compounds can be found in the paper by Rydfjord et al.^33^

### General Procedure for the Synthesis of [Carbonyl-^11^C]acyl Amidines

Aryl halide (9 μmol), benzamidine (2.0 equiv), triethylamine (4.0 equiv), and Pd(PPh_3_)_4_ (0.1 equiv) were transferred to a 950 μL oven-dried conical vial and dissolved in tetrahydrofuran (400 μL). After the vial was capped, the reaction mixture was sonicated and purged with N_2_ before being placed in the xenon system.^46^ The [^11^C]CO_2_ produced in the cyclotron was transferred to the xenon system in a stream of helium gas and concentrated on a CO_2_-trap immersed in liquid nitrogen. Heating the trap released [^11^C]CO_2_, which was reduced to [^11^C]CO over zinc heated to 400 °C. Residual [^11^C]CO_2_ was trapped on an Ascarite column, and [^11^C]CO was concentrated on a CO-trap immersed in liquid nitrogen. Before the CO-trap was heated, the carrier gas was changed from helium to xenon (>99.9%, 1.5 mL/min). The concentrated [^11^C]CO was transferred to the capped reaction vial through a transfer needle.

### Optimization of the Synthesis of [Carbonyl-^11^C]acyl Amidines

For the optimization experiments, different reaction parameters were used (see Table 1 and 2). After the completion of the reaction, the radioactivity was measured before the reaction vial was vented and purged. The radioactivity of the vented reaction vial was then measured. These radioactivity measurements were used to calculate the amount of decay-corrected [^11^C]CO trapped in the reaction mixture by dividing the decay-corrected radioactivity of the vented reaction vial with the radioactivity in the reaction vial after the reaction (see the Supporting Information for more details).

### Synthesis and Isolation of [Carbonyl-^11^C]acyl Amidines

For the synthesis and isolation of the [carbonyl-^11^C]acyl amidines, the radioactivity in the reaction vial was measured after collection of [^11^C]CO before the reaction at 120 °C for 5 or 10 min. After the reaction was complete, the reaction mixture was diluted with 400 μL of 60% acetonitrile (aq) and semipreparative HPLC purification was performed (semipreparative purification method, 10%–50% acetonitrile 0–15 min, 50%–80% acetonitrile 15–20 min, 80%–90% acetonitrile 20–20.5 min, 90% acetonitrile 20.5–25 min, 5 mL/min flow, and UV 254 nm). The radioactivity of the isolated ^11^C-labeled product fraction was measured, and a sample was taken for HPLC analysis of the radiochemical purity (RCP). Analysis was performed on either a Chromolith Performance RP-18e column or a Kinetex C18 column (analytical method, 10–90% acetonitrile 0–10 min, 90%–100% acetonitrile 10–10.5 min, 100% acetonitrile 10.5–13 min, 100–10% acetonitrile 13–13.5 min, 10% acetonitrile 13.5–15 min, 1 mL/min 0–10 min and 2 mL/min 10–15 min flow, and UV 254 nm). The radiochemical yield (RCY) is based on the amount of collected [^11^C]CO in the reaction vial and the amount of ^11^C-labeled product, the latter of which is decay-corrected to the time of the [^11^C]CO measurement. See the Supporting Information for more details and for radiochromatograms of the isolated products confirming their identity.

#### [Carbonyl-^11^C]4-methyl-*N*-(imino(phenyl)methyl)benzamide **3a**

Synthesized according to the general method using 4-iodotoluene, benzamidine, and conditions as in entry 1 of Table 1. Experiment 1: starting with 5.81 GBq of [^11^C]CO, 1.203 GBq of [^11^C]3a was isolated 34 min from the start of the reaction. Experiment 2: starting with 5.11 GBq of [^11^C]CO, 1.203 GBq of [^11^C]3a was isolated 31 min from the start of the reaction. Experiment 3: starting with 3.54 GBq of [^11^C]CO, 0.99 GBq of [^11^C]3a was isolated 29 min from the start of the reaction. Experiment 4 (5 min reaction time): starting with 4.49 GBq of [^11^C]CO, 0.57 GBq of [^11^C]3a was isolated 31 min from the start of the reaction. Experiment 5 (5 min reaction time): starting with 3.76 GBq of [^11^C]CO, 1.41 GBq of [^11^C]3a was isolated 26 min from the start of the reaction. Experiment 6 (5 min reaction time): starting with 3.63 GBq of [^11^C]CO, 1.07 GBq of [^11^C]3a was isolated 26 min from the start of the reaction. Analyzed with a Chromolith column, *t*_R_ = 3.7 min. RCP > 99% (10 min reaction time) and RCP > 99% (5 min reaction time).

Synthesized according to the general method using 4-bromotoluene, benzamidine, and conditions as in entry 5, Table 2. Experiment 1: starting with 3.03 GBq of [^11^C]CO, 0.62 GBq of [^11^C]3a was isolated 33 min from the start of the reaction. Experiment 2: starting with 3.04 GBq of [^11^C]CO, 0.92 GBq of [^11^C]3a was isolated 28 min from the start of the reaction. Analyzed with a Kinetex column, *t*_R_ = 8.1 min. RCP > 99%.

#### [Carbonyl-^11^C]4-methoxy-*N*-(imino(phenyl)methyl)benzamide **3b**

Synthesized according to the general method using 4-iodoanisole, benzamidine, and conditions as in entry 1 of Table 1. Experiment 1: starting with 3.14 GBq of [^11^C]CO, 0.99 GBq of [^11^C]3b was isolated 27 min from the start of the reaction. Experiment 2: starting with 2.85 GBq of [^11^C]CO, 0.90 GBq of [^11^C]3b was isolated 25 min from the start of the reaction. Analyzed with a Chromolith column, *t*_R_ = 2.9 min. RCP > 99%.

Synthesized according to the general method using 4-bromoanisole, benzamidine, and conditions as in entry 5 of Table 2. Experiment 1: starting with 2.05 GBq of [^11^C]CO, 0.54 GBq of [^11^C]3b was isolated 25 min from the start of the reaction. Experiment 2: starting with 2.97 GBq of [^11^C]CO, 0.67 GBq of [^11^C]3b was isolated 28 min from the start of the reaction. Analyzed with a Kinetex column, *t*_R_ = 7.4 min. RCP > 99%.

#### [Carbonyl-^11^C]4-acetyl-*N*-(imino(phenyl)methyl)benzamide **3c**

Synthesized according to the general method using 1-(4-iodophenyl)-ethan-1-one, benzamidine, and conditions as in entry 1 of Table 1. Experiment 1: starting with 2.95 GBq of [^11^C]CO, 0.98 GBq of [^11^C]3c was isolated 23 min from the start of the reaction. Experiment 2: starting with 2.55 GBq of [^11^C]CO, 0.83 GBq of [^11^C]3c was isolated 24 min from the start of the reaction. Analyzed with a Chromolith column, *t*_R_ = 4.8 min. RCP > 99%.

Synthesized according to the general method using 1-(4-bromophenyl)-ethan-1-one, benzamidine, and conditions as in entry 5 of Table 2. Experiment 1: starting with 4.48 GBq of [^11^C]CO, 0.90 GBq of [^11^C]3c was isolated 32 min from the start of the reaction. Experiment 2: starting with 2.57 GBq of [^11^C]CO, 0.61 GBq of [^11^C]3c was isolated 28 min from the start of the reaction. Analyzed with a Kinetex column, *t*_R_ = 7.1 min. RCP > 93%.

#### [Carbonyl-^11^C]4-bromo-*N*-(imino(phenyl)methyl)benzamide **3e**

Synthesized according to the general method using 1-bromo-4-iodobenzene, benzamidine, and conditions as in entry 1 of Table 1. Experiment 1: starting with 2.18 GBq of [^11^C]CO, 0.57 GBq of [^11^C]3e was isolated 26 min from the start of the reaction. Experiment 2: starting with 2.85 GBq of [^11^C]CO, 0.70 GBq of [^11^C]3e was isolated 27 min from the start of the reaction. Analyzed with a Chromolith column, *t*_R_ = 6.6 min. RCP > 99%.

#### [Carbonyl-^11^C]-*N*-(imino(phenyl)methyl)-5 methylthiophene-2-carboxamide **3f**

Synthesized according to the general method using 2-iodo-5-methylthiophene, benzamidine, and conditions as in entry 1 of Table 1. Experiment 1: starting with 3.37 GBq of [^11^C]CO, 1.14 GBq of [^11^C]3f was isolated 26 min from the start of the reaction. Experiment 2: starting with 2.88 GBq of [^11^C]CO, 0.97 GBq of [^11^C]3f was isolated 28 min from the start of the reaction. Analyzed with a Chromolith column, *t*_R_ = 5.4 min. RCP > 99%.

#### [Carbonyl-^11^C]-*N*-(cyclohexyl(imino)methyl)-4-methylbenzamide **3g**

Synthesized according to the general method using 4-iodotoluene, cyclohexyl amidine, and conditions as in entry 1 of Table 1. Experiment 1: starting with 4.83 GBq of [^11^C]CO, 1.61 GBq of [^11^C]3g was isolated 30 min from the start of the reaction. Experiment 2: starting with 4.71 GBq of [^11^C]CO, 0.99 GBq of [^11^C]3g was isolated 30 min from the start of the reaction. Experiment 3: starting with 2.52 GBq of [^11^C]CO, 0.54 GBq of [^11^C]3g was isolated 30 min from the start of the reaction. Experiment 4: starting with 2.02 GBq of [^11^C]CO, 0.48 GBq of [^11^C]3g was isolated 28 min from the start of the reaction. Analyzed with a Chromolith column, *t*_R_ = 3.5 min. RCP > 98%.

Synthesized according to the general method using 4-bromotoluene, cyclohexyl amidine, and conditions as in entry 5 of Table 2. Experiment 1: starting with 2.83 GBq of [^11^C]CO, 0.41 GBq of [^11^C]3g was isolated 33 min from the start of the reaction. Experiment 2: starting with 3.97 GBq of [^11^C]CO, 0.72 GBq of [^11^C]3g was isolated 32 min from the start of the reaction. Analyzed with a Kinetex column, *t*_R_ = 7.6 min. RCP > 99%.

#### [Carbonyl-^11^C]-*N*-(imino(4-methoxyphenyl)methyl)-4-methylbenzamide **3h**

Synthesized according to the general method using 4-iodotoluene, 4-methoxybenzamidine, and conditions as in entry 1 of Table 1. Experiment 1: starting with 2.99 GBq of [^11^C]CO, 0.80 GBq of [^11^C]3h was isolated 26 min from the start of the reaction. Experiment 2: starting with 2.55 GBq of [^11^C]CO, 0.68 GBq of [^11^C]3h was isolated 26 min from the start of the reaction. Analyzed with a Kinetex column, *t*_R_ = 5.9 min (flow 2 mL/min 0–15 min). RCP > 99%.

Synthesized according to the general method using 4-bromotoluene, 4-methoxybenzamidine, and conditions as in entry 5 of Table 2. Experiment 1: starting with 2.37 GBq of [^11^C]CO, 0.50 GBq of [^11^C]3h was isolated 28 min from the start of the reaction. Experiment 2: starting with 3.37 GBq of [^11^C]CO, 0.62 GBq of [^11^C]3h was isolated 32 min from the start of the reaction. Analyzed with a Kinetex column, *t*_R_ = 8.0 min. RCP > 99%.

#### [Carbonyl-^11^C]-*N*-(imino(4-chlorophenyl)methyl)-4-methylbenzamide **3i**

Synthesized according to the general method using 4-iodotoluene, 4-chlorobenzamidine, and conditions as in entry 1 of Table 1. Experiment 1: starting with 2.49 GBq of [^11^C]CO, 0.56 GBq of [^11^C]3i was isolated 27 min from the start of the reaction. Experiment 2: starting with 3.13 GBq of [^11^C]CO, 0.68 GBq of [^11^C]3i was isolated 29 min from the start of the reaction. Analyzed with a Chromolith column, *t*_R_ = 8.6 min. RCP > 99%.

#### [Carbonyl-^11^C]-*N*-(imino(4-(trifluoromethyl)phenyl)methyl)-4-methylbenzamide **3j**

Synthesized according to the general method using 4-iodotoluene, 4-(trifluoromethyl)benzamidine, and conditions as in entry 1 of Table 1. Experiment 1: starting with 2.44 GBq of [^11^C]CO, 0.30 GBq of [^11^C]3j was isolated 29 min from the start of the reaction. Experiment 2: starting with 1.98 GBq of [^11^C]CO, 0.35 GBq of [^11^C]3j was isolated 27 min from the start of the reaction. Analyzed with a Kinetex column, *t*_R_ = 9.6 min. RCP > 98%.

Synthesized according to the general method using 4-bromotoluene, 4-methoxybenzamidine, and conditions as in entry 5 of Table 2. Experiment 1: starting with 2.48 GBq of [^11^C]CO, 0.36 GBq of [^11^C]3j was isolated 31 min from the start of the reaction. Experiment 2: starting with 3.34 GBq of [^11^C]CO, 0.58 GBq of [^11^C]3j was isolated 30 min from the start of the reaction. Analyzed with a Kinetex column, *t*_R_ = 9.3 min. RCP > 95%.

#### [Carbonyl-^11^C]-*N*-(imino(2-methyl-1H-indol-3-yl)methyl)-4-methylbenzamide **3k**

Synthesized according to the general method using 4-iodotoluene, 1*H*-indole-3-amidine, and conditions as in entry 1 of Table 1. Experiment 1: starting with 1.94 GBq of [^11^C]CO, 0.60 GBq of [^11^C]3k was isolated 27 min from the start of the reaction. Experiment 2: starting with 4.03 GBq of [^11^C]CO, 1.08 GBq of [^11^C]3k was isolated 31 min from the start of the reaction. Analyzed with a Kinetex column, *t*_R_ = 5.4 min. RCP > 99%.

### General Procedure for the Synthesis of ^11^C-labeled Oxadiazoles

The ^11^C-labeled oxadiazoles were synthesized following the general procedure for the synthesis of [carbonyl-^11^C]acyl amidines, but the reaction temperature was increased to 150 °C after 4 min reaction. After the completion of the synthesis of [carbonyl-^11^C]acyl amidines, hydroxylamine hydrochloride (7 equiv) dissolved in 150 μL of 50% acetic acid (aq) was added to the reaction vial. The reaction vial was then heated at 150 °C for 5 min.

### Optimization of the Synthesis of ^11^C-Labeled Oxadiazoles

The time for increasing the reaction temperature in the synthesis of [carbonyl-^11^C]acyl amidines was evaluated during optimization as well as the cyclization time and the choice of solvent. For the optimization experiments, the radioactivity was measured in the reaction vial after the [carbonyl-^11^C]acyl amidine synthesis. The vial was vented before the addition of the hydroxylamine hydrochloride solution. The reaction was heated at 150 °C for either 5 or 10 min. After completion of the reaction, the reaction vial was again vented and purged before the radioactivity in the reaction vial was measured.

### Synthesis and Isolation of ^11^C-Labeled Oxadiazoles

The synthesis and isolation of ^11^C-labeled oxadiazoles follows the general procedure outlined above. After the completion of the reaction, the reaction mixture was diluted with 400 μL of 60% acetonitrile (aq), and semipreparative HPLC purification was performed (semipreparative purification method, 70%–100% acetonitrile 0–5 min, 100% acetonitrile 5–20 min, 5 mL/min flow, and UV 254 nm). For [^11^C]DDO-7263, the following gradient was used: 30%–80% acetonitrile 0–10 min, 80%–100% 10–10.5 min, 100% acetonitrile 10.5–20 min, 5 mL/min flow, and UV 254 nm. The radioactivity of the isolated ^11^C-product fraction was measured, and a sample was taken for HPLC analysis of the RCP. Analysis was performed on a Kinetex C18 column (analytical method, 30–90% acetonitrile 0–10 min, 90%–100% acetonitrile 10–10.5 min, 100% acetonitrile 10.5–13 min, 100–10% acetonitrile 13–13.5 min, 10% acetonitrile 13.5–15 min, 1 mL/min flow, and UV 254 nm). The RCY was calculated in the same way as for the [carbonyl-^11^C]acyl amidines. See the Supporting Information for more details and for radiochromatograms confirming the identities of the isolated products.

#### 3-Phenyl-5-(4-tolyl)-1,2,4-(5-^11^C)oxadiazole **4a**

Synthesized according to the general method using 4-iodotoluene, benzamidine, and conditions as in entry 2 of Table 3. Experiment 1: starting with 1.64 GBq of [^11^C]CO, 0.13 GBq of [^11^C]4a was isolated 45 min from the start of the reaction. Experiment 2: starting with 2.14 GBq of [^11^C]CO, 0.38 GBq of [^11^C]4a was isolated 31 min from the start of the reaction. Experiment 3: starting with 3.44 GBq of [^11^C]CO, 0.10 GBq of [^11^C]4a was isolated 70 min from the start of the reaction (there were problems with the synthetic equipment, hence the long synthesis time). Experiment 4: starting with 24.7 GBq of [^11^C]CO, 2.61 GBq of [^11^C]4a was isolated 35 min from the start of the reaction. Experiment 5: starting with 16.9 GBq of [^11^C]CO, 1.28 GBq of [^11^C]4a was isolated 33 min from the start of the reaction. The molar activity of **4a** in experiments 4 and 5 was 512 and 401 GBq/μmol, respectively. Analyzed with a Kinetex column, *t*_R_ = 9.4 min. RCP > 99%.

Synthesized according to the general method using 4-bromotoluene, benzamidine, and conditions as in entry 1 of Table 4. Experiment 1: starting with 3.26 GBq of [^11^C]CO, 0.28 GBq of [^11^C]4a was isolated 39 min from the start of the reaction. Experiment 2: starting with 2.55 GBq of [^11^C]CO, 0.29 GBq of [^11^C]4a was isolated 36 min from the start of the reaction. Analyzed with a Kinetex column, *t*_R_ = 10.2 min. RCP > 99%.

#### 3-Phenyl-5-(4-methoxyphenyl)-1,2,4-(5-^11^C)oxadiazole **4b**

Synthesized according to the general method using 4-iodotoluene, 4-methoxybenzamidine, and conditions as in entry 2 of Table 3. Experiment 1: starting with 2.55 GBq of [^11^C]CO, 0.60 GBq of [^11^C]4b was isolated 30 min from the start of the reaction. Experiment 2: starting with 3.04 GBq of [^11^C]CO, 0.36 GBq of [^11^C]4b was isolated 29 min from the start of the reaction. Experiment 3: starting with 3.05 GBq of [^11^C]CO, 0.39 GBq of [^11^C]4b was isolated 31 min from the start of the reaction. Analyzed with a Kinetex column, *t*_R_ = 8.5 min. RCP > 99%.

Synthesized according to the general method using 4-bromotoluene, 4-methoxybenzamidine, and conditions as in entry 1 of Table 4. Experiment 1: starting with 2.61 GBq of [^11^C]CO, 0.37 GBq of [^11^C]4b was isolated 33 min from the start of the reaction. Experiment 2: starting with 2.12 GBq of [^11^C]CO, 0.23 GBq of [^11^C]4b was isolated 32 min from the start of the reaction. Analyzed with a Kinetex column, *t*_R_ = 9.7 min. RCP > 99%.

#### 3-Phenyl-5-([4-trifluoromethyl]phenyl)-1,2,4-(5-^11^C)oxadiazole **4c**

Synthesized according to the general method using 4-iodotoluene, 4-(trifluoromethyl)benzamidine, and conditions as in entry 2 of Table 3. Experiment 1: starting with 2.89 GBq of [^11^C]CO, 0.06 GBq of [^11^C]4c was isolated 41 min from the start of the reaction. Experiment 2: starting with 4.87 GBq of [^11^C]CO, 0.27 GBq of [^11^C]4c was isolated 33 min from the start of the reaction. Analyzed with a Kinetex column, *t*_R_ = 9.6 min. RCP > 98%.

Synthesized according to the general method using 4-bromotoluene, 4-(trifluoromethyl)benzamidine, and conditions as in entry 1 of Table 4. Experiment 1: starting with 2.35 GBq of [^11^C]CO, 0.09 GBq of [^11^C]4c was isolated 36 min from the start of the reaction. Experiment 2: starting with 2.44 GBq of [^11^C]CO, 0.08 GBq of [^11^C]4c was isolated 35 min from the start of the reaction. Analyzed with a Kinetex column, *t*_R_ = 10.4 min. RCP > 87%.

#### 5-(3,4-Difluorophenyl)-3-(6-methylpyridin-3-yl)-1,2,4-oxadiazole-5-^11^C [^11^C]DDO-7263

Synthesized according to the general method using 1,2-difluoro-4-iodobenzene, 6-methylpyridine-3-carboxamidine, and conditions as in entry 2 of Table 3. Experiment 1: starting with 0.949 GBq of [^11^C]CO, 0.074 GBq of [^11^C]DDO-7263 was isolated 39 min from the start of the reaction. Analyzed with a Kinetex column, *t*_R_ = 7.1 min. RCP 97%.

## Supporting Information Available

Details about calculations, standard curves and data for the molar activity determination, and HPLC chromatograms (UV and radio signals) for all isolated compounds for determination of RCP and identity (PDF)
