# Supplementary material for: Palladium-Mediated Synthesis of [Carbonyl-11C]acyl Amidines from Aryl Iodides and Aryl Bromides and Their One-Pot Cyclization to 11C-Labeled Oxadiazoles
Source: J Org Chem. 2022 Dec 13;88(8):5118–26. doi: 10.1021/acs.joc.2c02102 (PMC10127284; doi:10.1021/acs.joc.2c02102)

Supporting information

Palladium-mediated synthesis of [carbonyl-^{11}C]acyl amidines from aryl iodides and aryl bromides and their one-pot cyclization to ^{11}C-labelled oxadiazoles

Jonas Rydfjord, Silav Al-Bazaz and Sara Roslin*

Department of Medicinal Chemistry, Uppsala University, BMC Box 574, SE-751 23 Uppsala, Sweden

*Correspondence: Sara Roslin, Department of Medicinal Chemistry, Uppsala University, BMC Box 574, SE-751 23 Uppsala, Sweden. Email: sara.roslin@akademiska.se

Content

Calculations and definitions S2

Calibration curve and molar activity determination...S3

HPLC-chromatogram of isolated ^{11}C-labelled compounds S4

Calculations and definitions

[^{11}C]CO-conversion

The [^{11}C]CO-conversion is the measurement of [^{11}C]CO incorporated into non-volatile compounds. It was calculated from the radioactivity measurement of the reaction vial after completion of the reaction (A2) and after venting and purging of the reaction vial (A3). The A3 measurement has been decay-corrected (d.c.) to the time-point of A2 measurement.

$$\text{Conversion} = \frac{\text{A3 (d.c.)}}{A2}$$

Product selectivity and product identity

The product selectivity is the percentage of formed ^{11}C-labelled product and is based on a HPLC analysis of the reaction mixture and known retention time of the unlabelled product, confirming the identity of the formed ^{11}C-labelled product.

Radiochemical yield

The radiochemical yield (RCY) is based on a measurement of the radioactivity in the isolated ^{11}C-labelled product (A4) and the amount of [^{11}C]CO in the reaction vial, a measurement made prior to start of the reaction (A1). The A4 measurement has been decay-corrected to the time-point of A1 measurement.

$$RCY = \frac{A4\ (d.c.)}{A1}$$

An estimation of the radiochemical yield is calculated from the [^{11}C]CO-conversion and the product selectivity and thus gives the non-isolated radiochemical yield.

$$Non - isolated\ RCY = Conversion \times Product\ selectivity$$

Radiochemical purity

The radiochemical purity is analyzed with HPLC of an aliquot from the isolated ^{11}C-labelled product fraction.

Calibration curve and molar activity determination

A calibration curve for **4a** (3-phenyl-5-(*p*-tolyl)-(5-^{11}C)-1,2,4-oxadiazole) was prepared using six concentrations; 0.25, 0.5, 1.0, 5.0, 20.0 and 50.0 µg/mL. 50 µL was injected. Analysis was performed at 254 nm and a calibration curve was constructed (Figure S1). The molar activity was determined from two experiment starting with roughly 20 µAh irradiation and the equation derived from the calibration curve (Table S1).

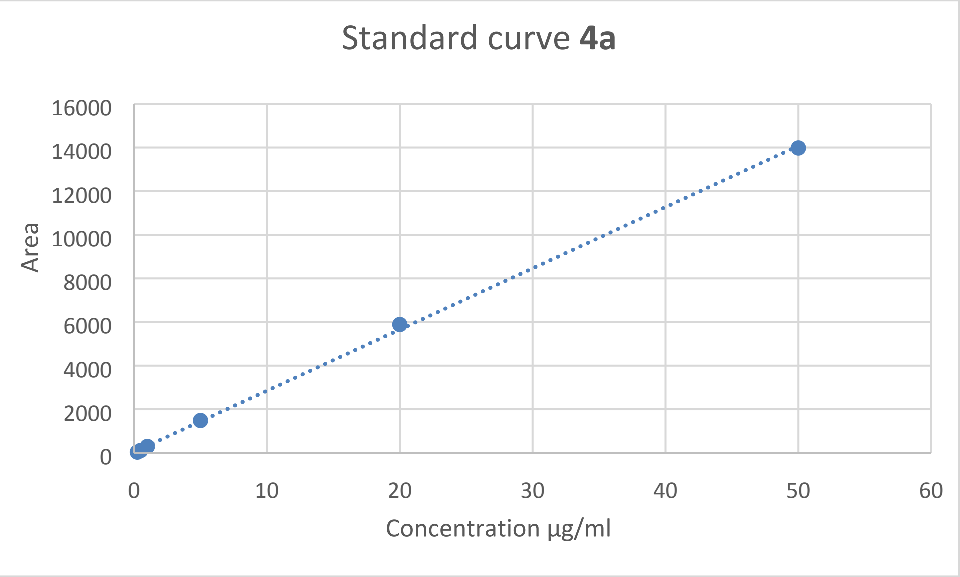

Figure S1. Calibration curve for **4a**.

Table S1. Determination of molar activity.

Exp.	Area	Concentration (µg/mL)	Volume (mL)	Mass (µg)	Amount (µmol)	Activity (GBq)	Molar activity (GBq/µmol)
**1**	159.161	0.42	2.9	1.21	0.0051	2.61	512
**2**	149.881	0.38	2.0	0.77	0.0032	1.28	401

HPLC-chromatogram of isolated ^{11}C-labelled compounds

Red signal = Radio chromatogram

Blue signal = UV chromatogram at 254 nm

The presented chromatograms are analyses of an aliquot of the isolated product to which unlabelled reference compound has been added, to confirm the identity of the isolated product.

[Carbonyl-^{11}C]4-methyl-N-(imino(phenyl)methyl)benzamide ***3a***

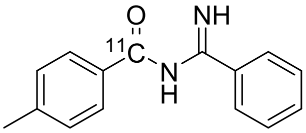

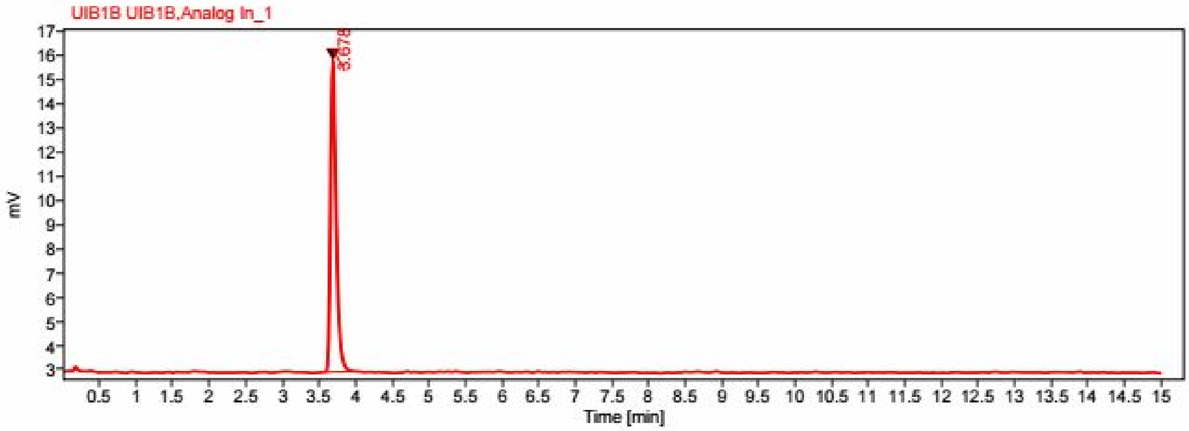

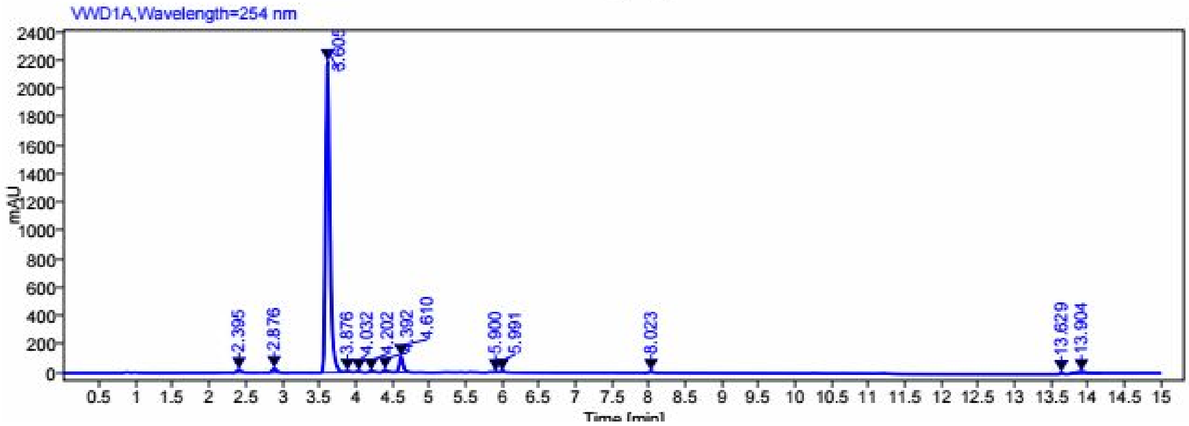

[Carbonyl-^{11}C]4-methoxy-N-(imino(phenyl)methyl)benzamide ***3b***

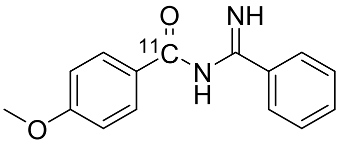

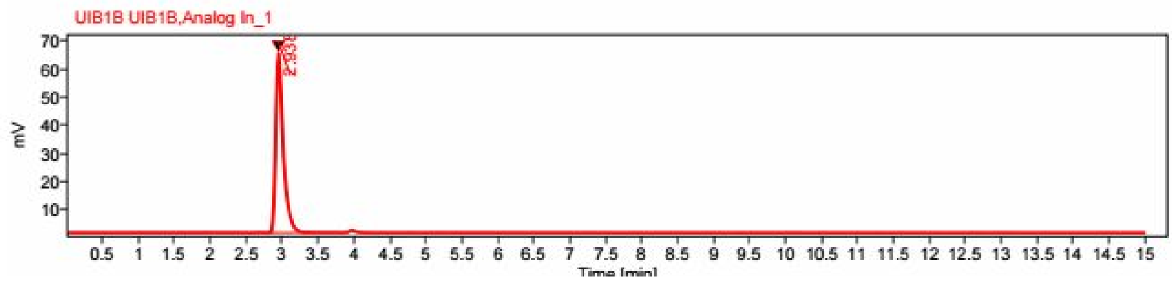

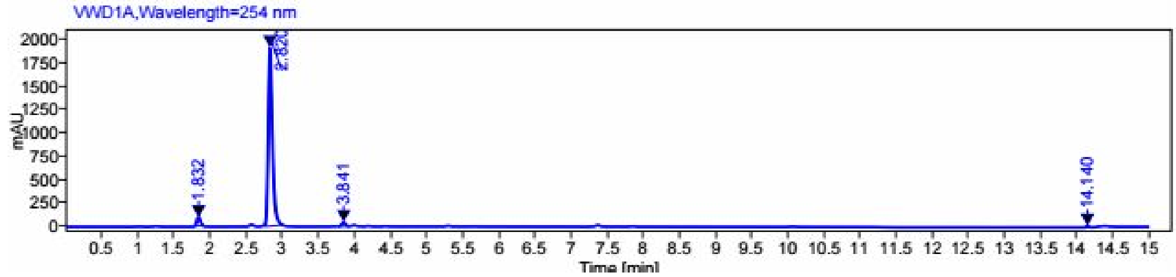

[Carbonyl-^{11}C]4-acetyl-N-(imino(phenyl)methyl)benzamide ***3c***

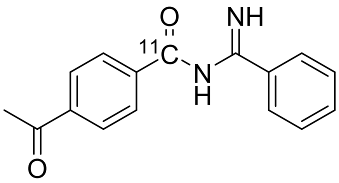

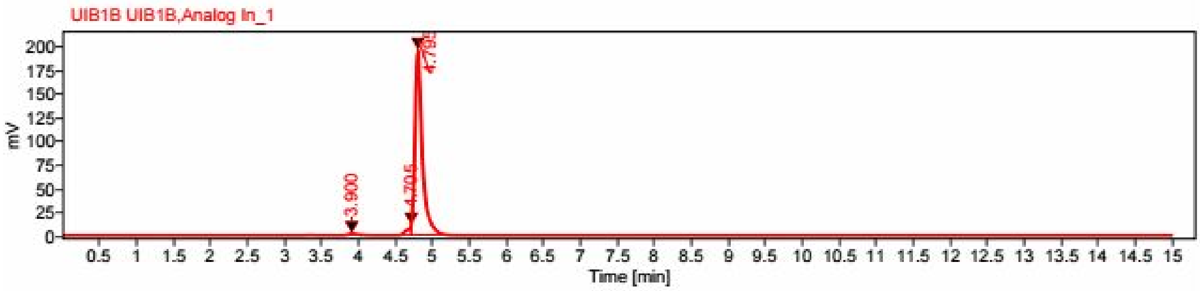

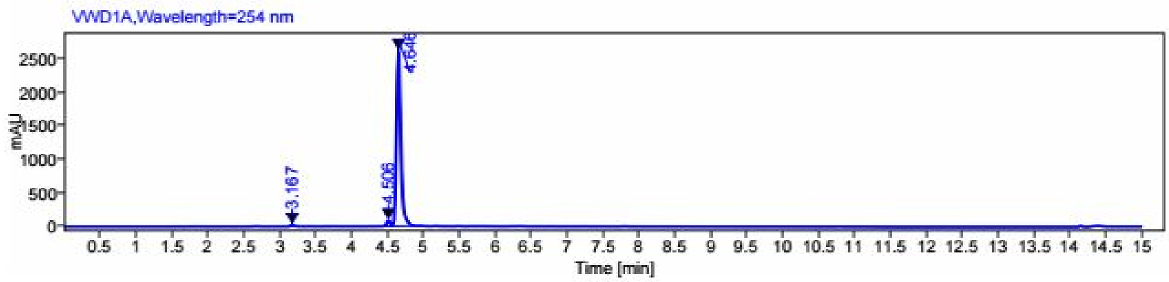

[Carbonyl-^{11}C]4-bromo-N-(imino(phenyl)methyl)benzamide ***3e***

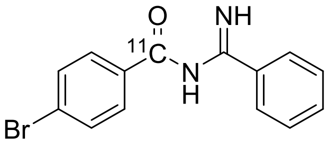

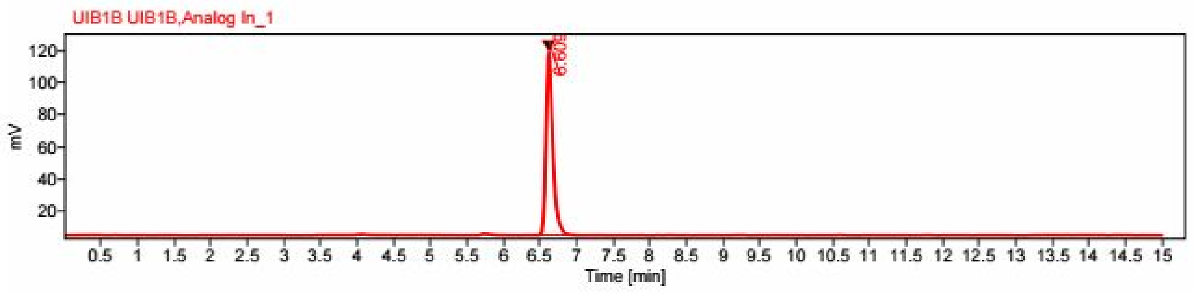

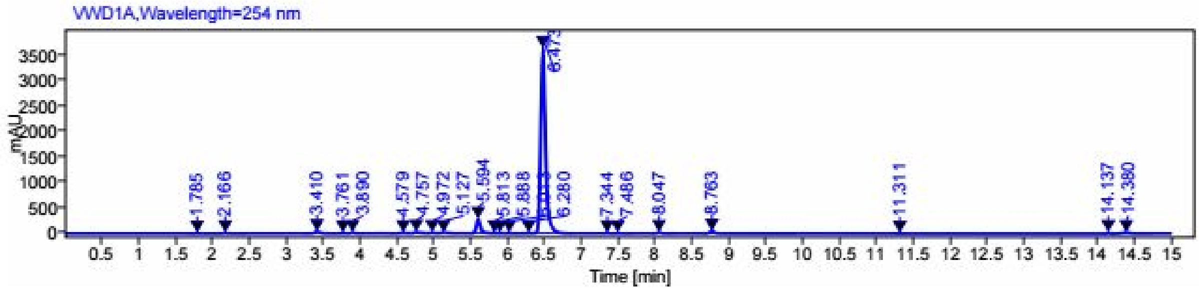

[Carbonyl-^{11}C]-N-(imino(phenyl)methyl)-5 methylthiophene-2-carboxamide ***3f***

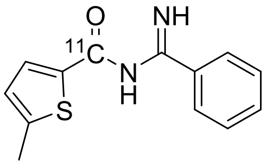

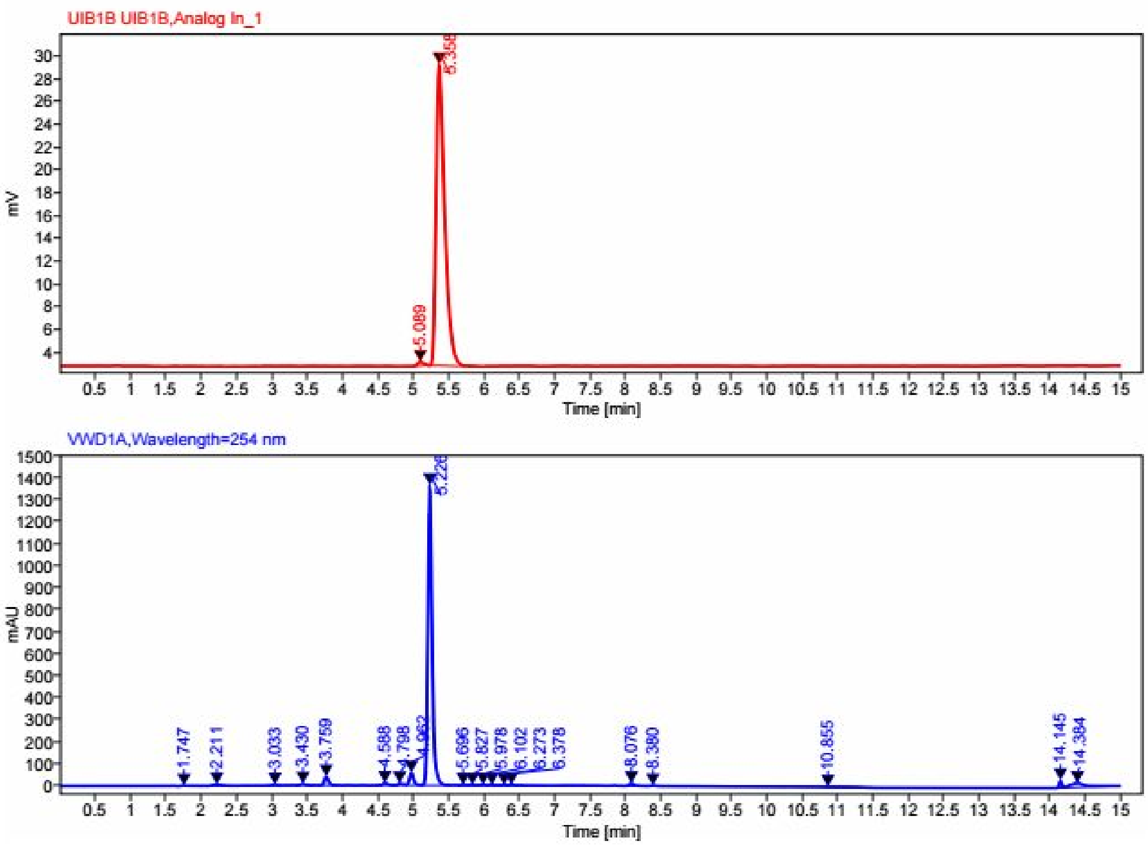

[Carbonyl-^{11}C]-N-(cyclohexyl(imino)methyl)-4-methylbenzamide ***3g***

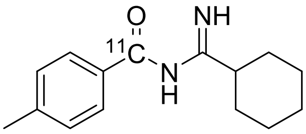

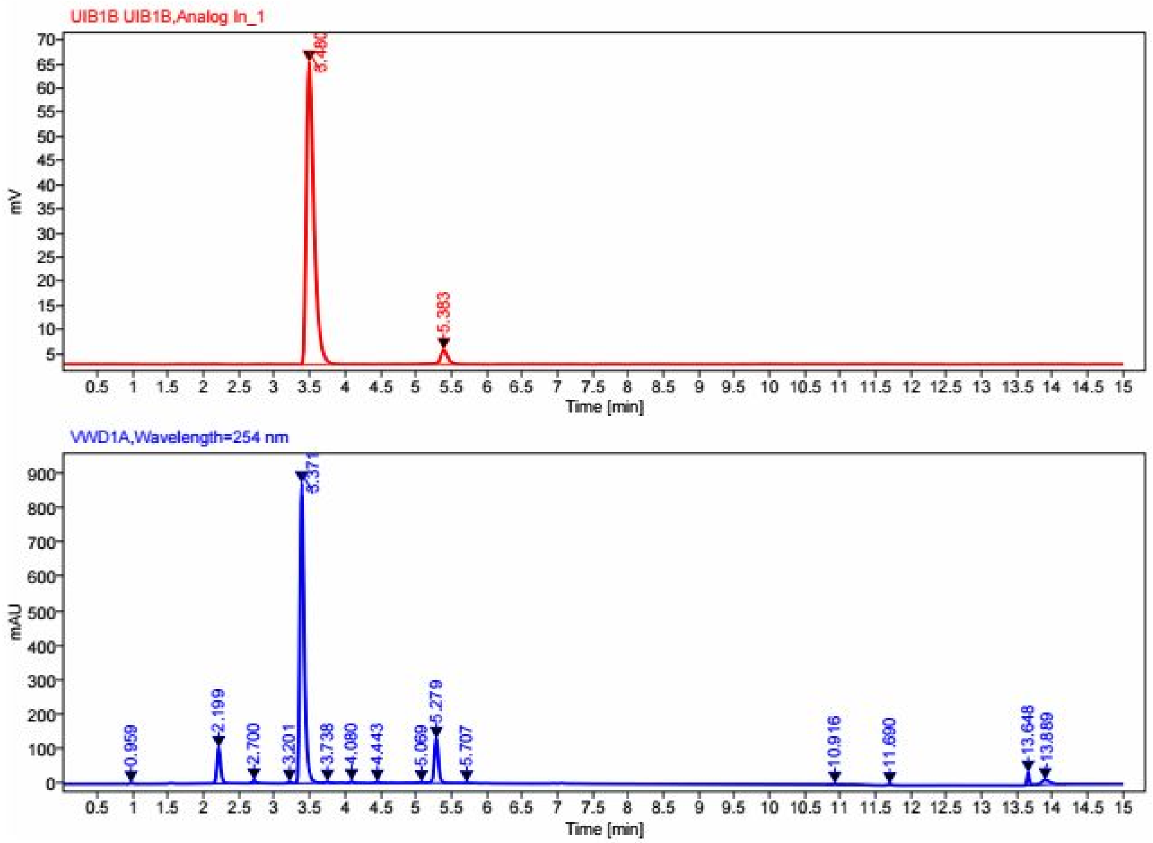

[Carbonyl-^{11}C]-N-(imino(4-methoxyphenyl)methyl)-4-methylbenzamide ***3h***

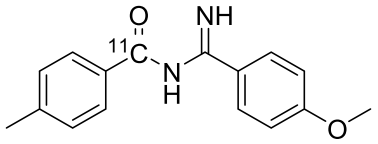

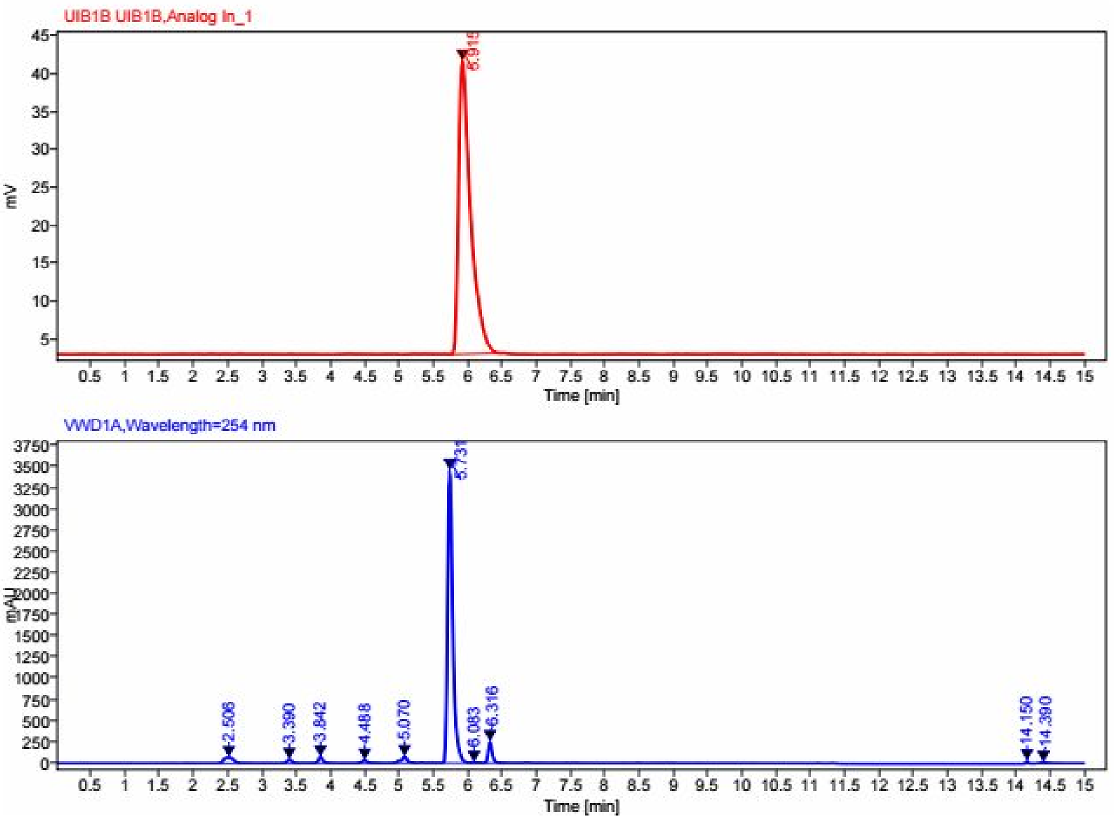

[Carbonyl-^{11}C]-N-(imino(4-chlorophenyl)methyl)-4-methylbenzamide ***3i***

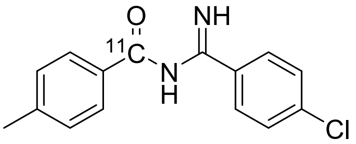

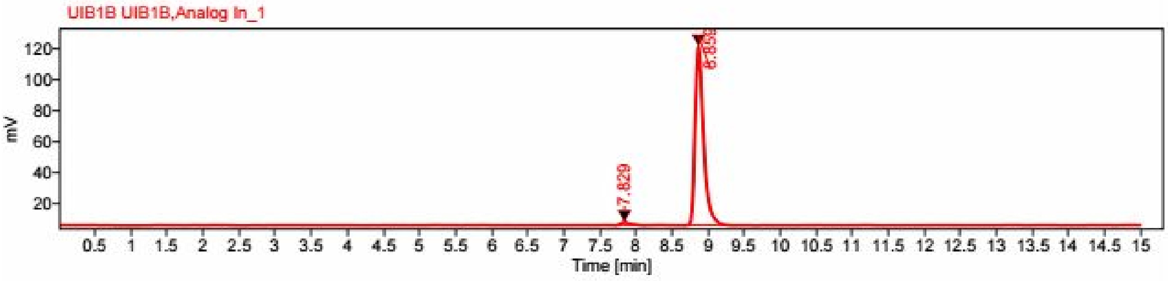

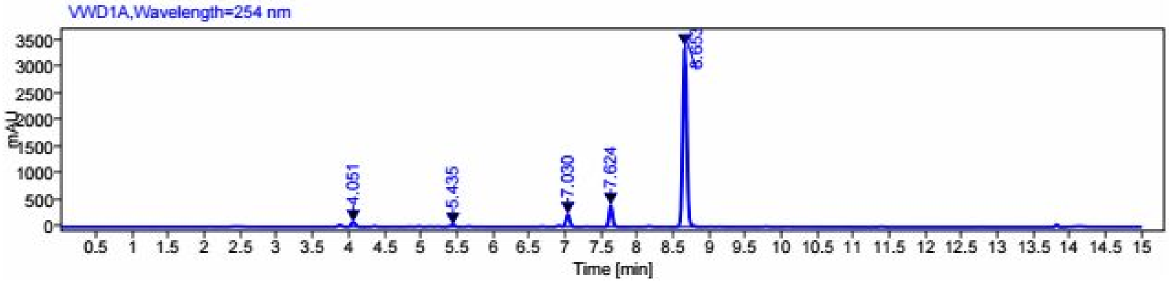

[Carbonyl-^{11}C]-N-(imino(4-(trifluoromethyl)phenyl)methyl)-4-methylbenzamide ***3j***

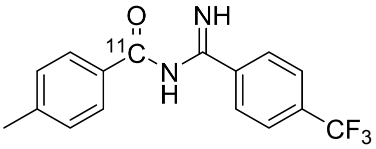

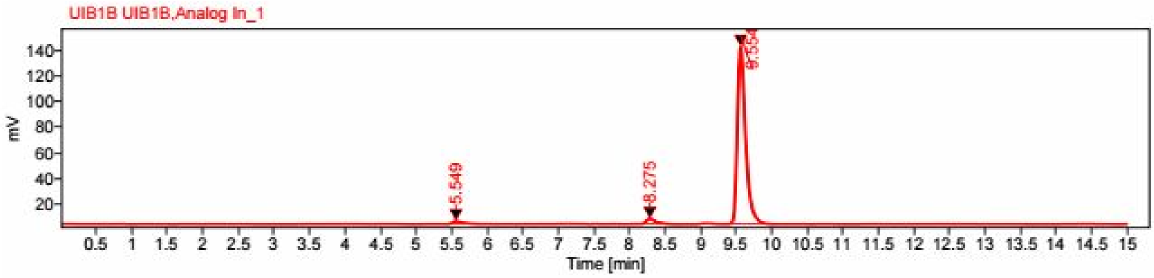

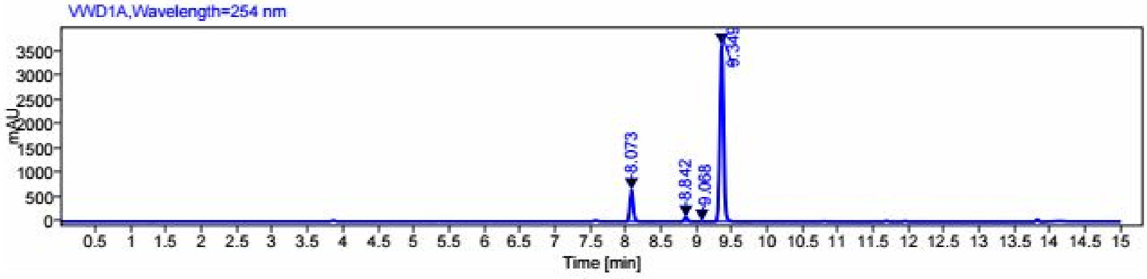

[Carbonyl-^{11}C]-N-(imino(2-methyl-1H-indol-3-yl)methyl)-4-methylbenzamide ***3k***

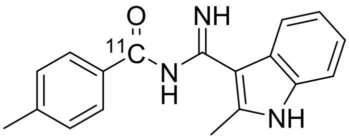

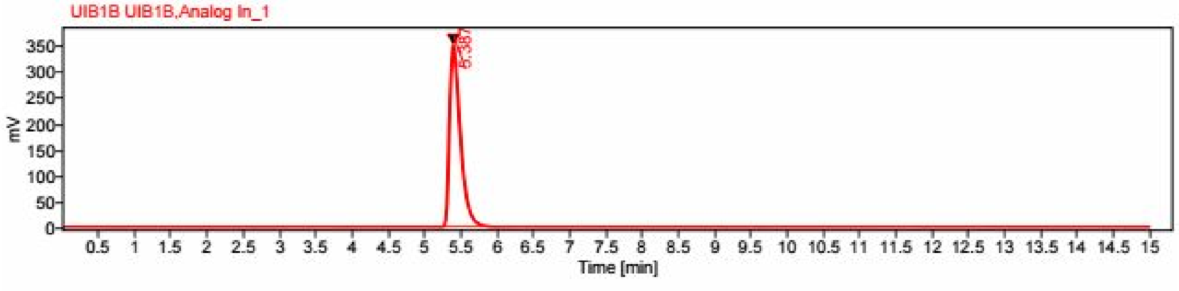

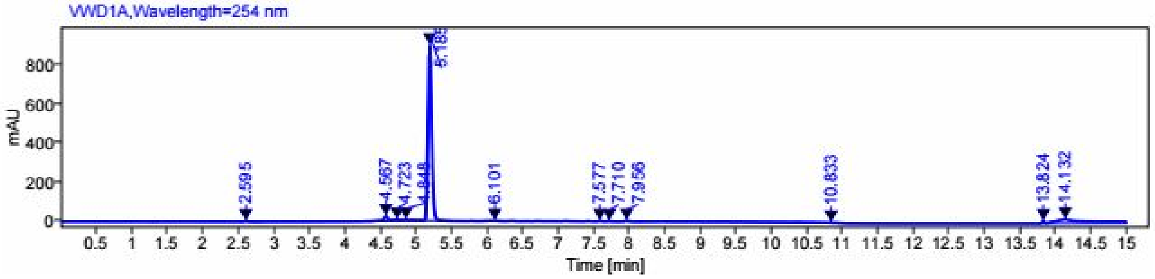

3-Phenyl-5-(4-tolyl)-1,2,4-(5-^{11}C)oxadiazole ***4a***

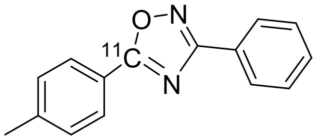

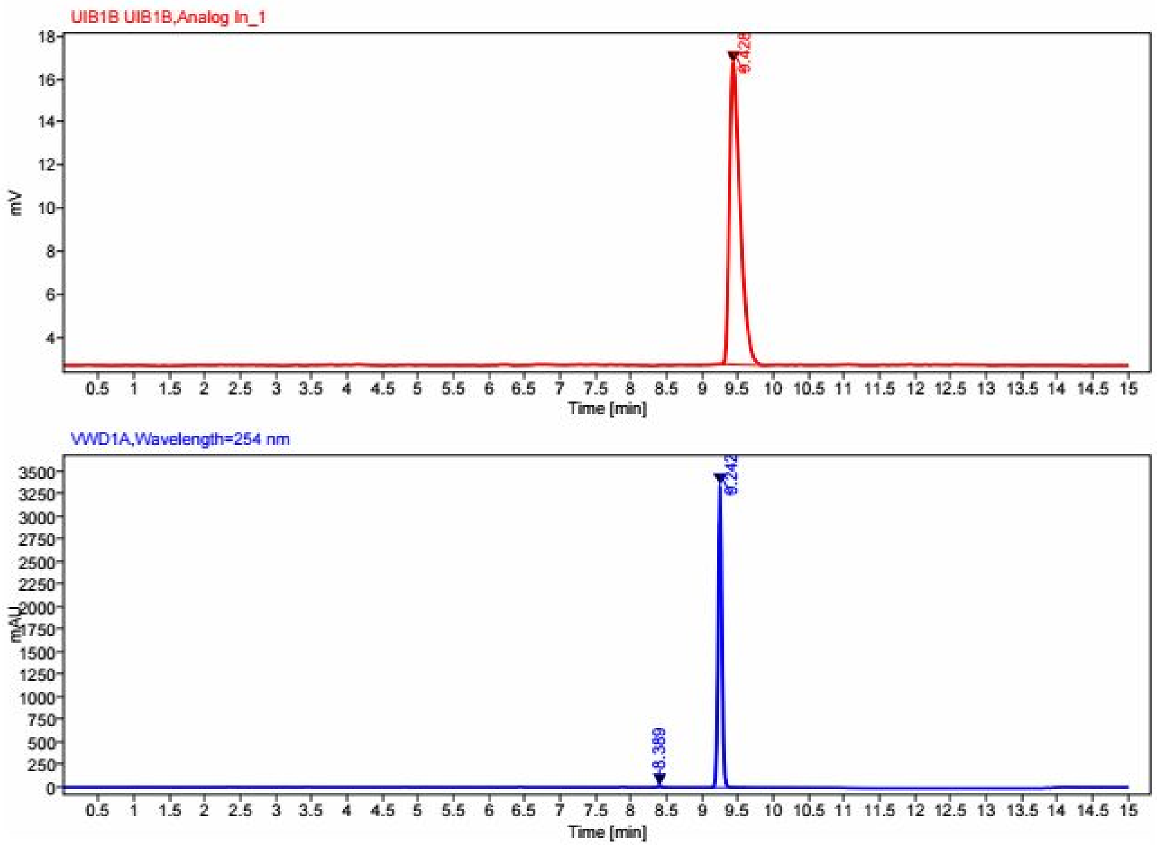

3-Phenyl-5-(4-methoxyphenyl)-1,2,4-(5-^{11}C)oxadiazole ***4b***

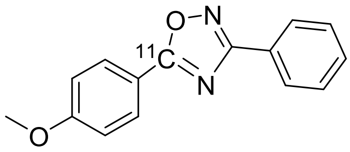

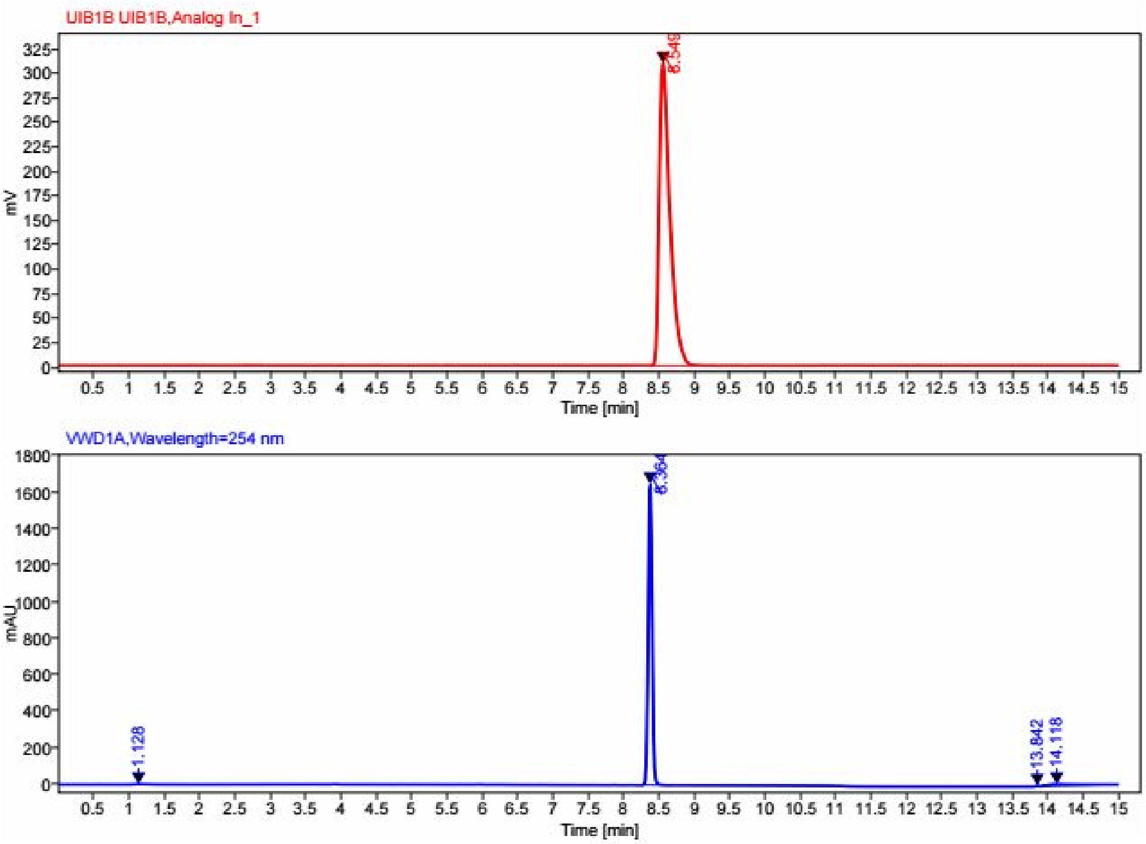

*3-Phenyl-5-([4-trifluoromethyl]phenyl)-1,2,4-(5-^{11}C)oxadiazole **4c***

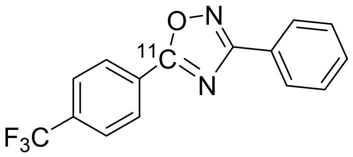

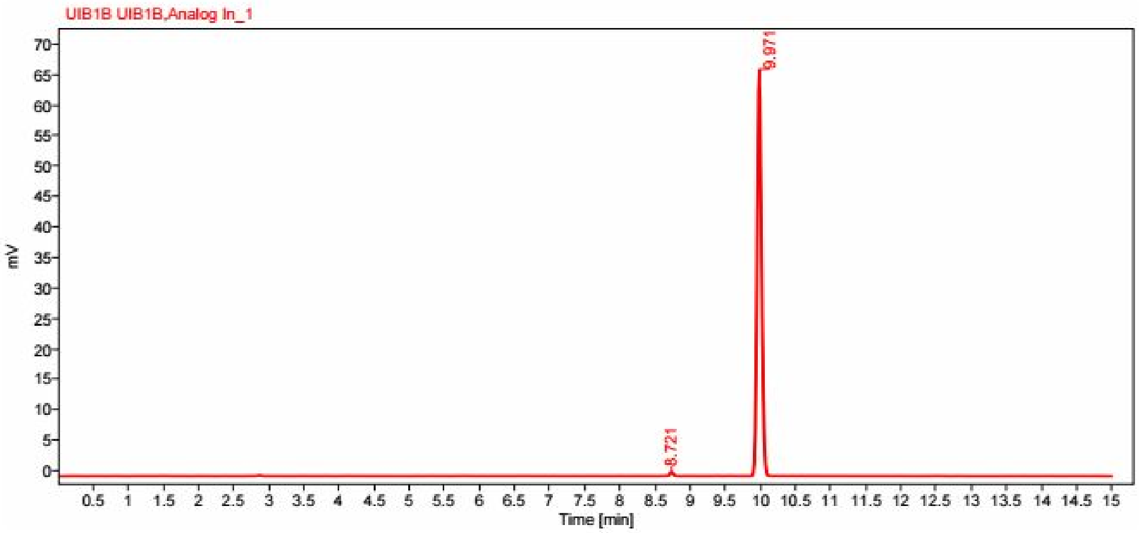

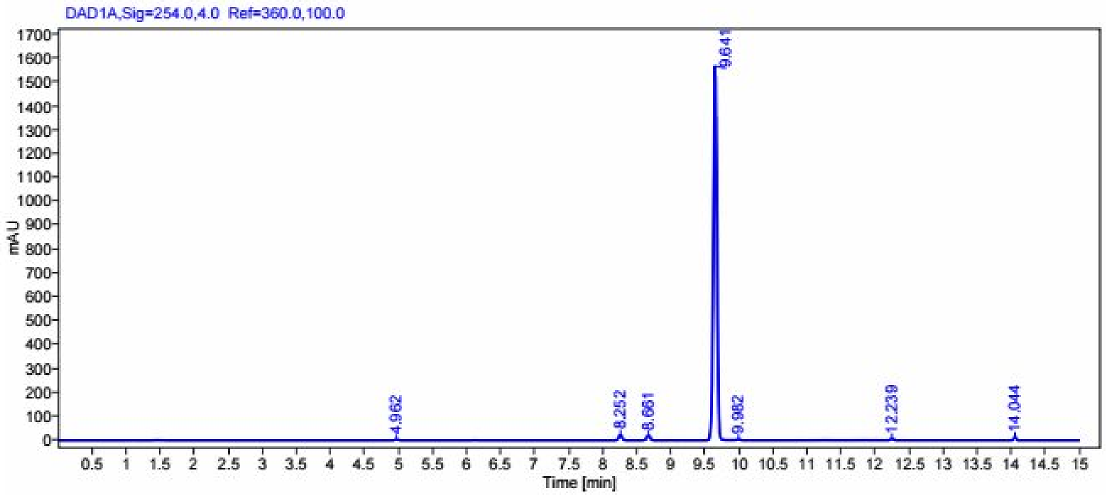

5-(3,4-difluorophenyl)-3-(6-methylpyridin-3-yl)-1,2,4-oxadiazole-5-^{11}C [^{11}C]DDO-7263

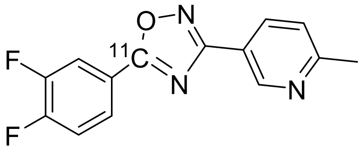

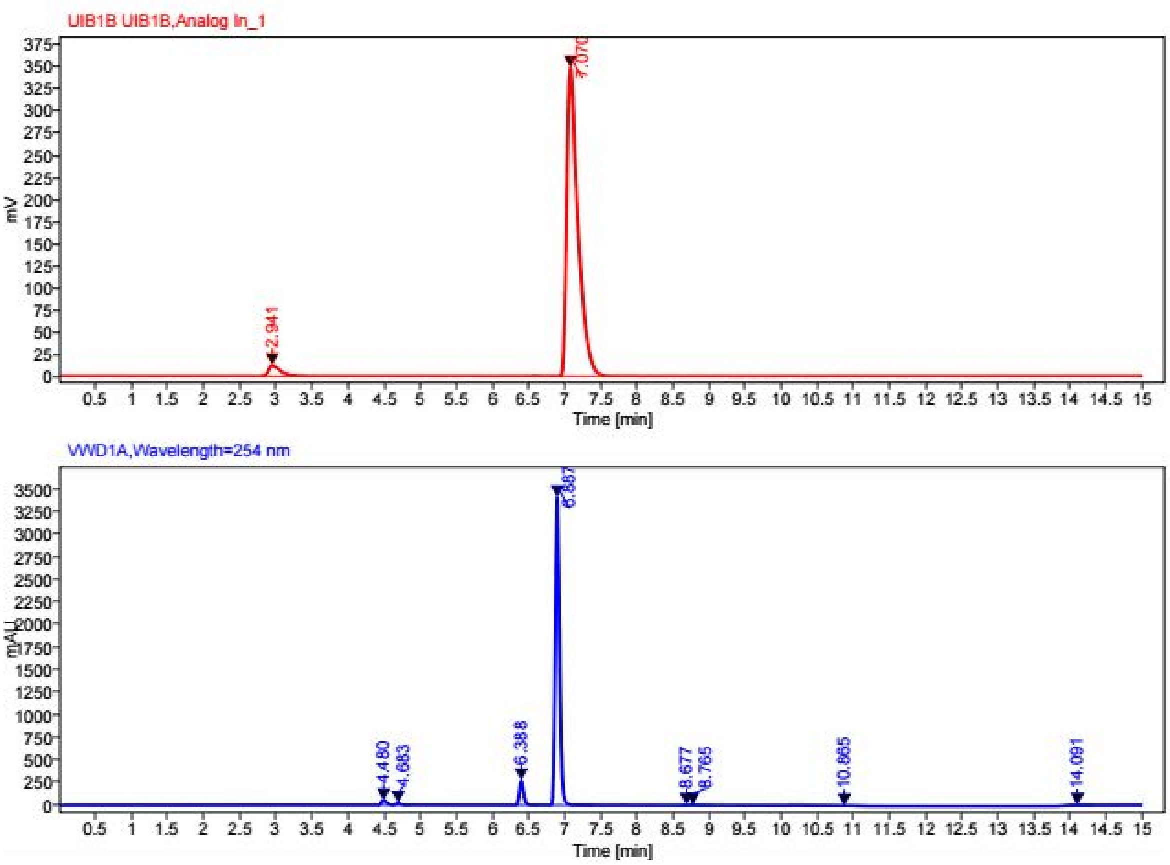

Supplement: Supplementary file 1 — jo2c02102_si_001.pdf [file jo2c02102_si_001.pdf]
